# NK105, a paclitaxel-incorporating micellar nanoparticle, is a more potent radiosensitising agent compared to free paclitaxel

**DOI:** 10.1038/sj.bjc.6603311

**Published:** 2006-08-08

**Authors:** T Negishi, F Koizumi, H Uchino, J Kuroda, T Kawaguchi, S Naito, Y Matsumura

**Affiliations:** 1 Investigative Treatment Division, Research Center for Innovative Oncology, National Cancer Center Hospital East, 6-5-1 Kashiwanoha, Kashiwa, Chiba 277-8577, Japan; 2 Department of Urology, Graduate School of Medical Sciences, Kyushu University, 3-1-1 Maidashi, Higashi-ku, Fukuoka, Fukuoka 812-8582, Japan; 3 Department of Anatomy and Histology, Fukushima Medical University School of Medicine, 1-Hikariga-oka, Fukushima, Fukushima 960-1247, Japan

**Keywords:** paclitaxel, NK105, radiosensitiser, polymer micelle, drug delivery system

## Abstract

NK105 is a micellar nanoparticle formulation designed to enhance the delivery of paclitaxel (PTX) to solid tumours. It has been reported to exert antitumour activity *in vivo* and to have reduced neurotoxicity as compared to that of free PTX. The purpose of this study was to investigate the radiosensitising effect of NK105 in comparison with that of PTX. Lewis lung carcinoma (LLC)-bearing mice were administered a single intravenous (i.v.) injection of PTX or NK105; 24 h after the drug administration, a proportion of the mice received radiation to the tumour site or lung fields. Then, the antitumour activity and lung toxicity were evaluated. In one subset of mice, the tumours were excised and specimens were prepared for analysis of the cell cycle distribution by flow cytometry. Combined NK105 treatment with radiation yielded significant superior antitumour activity as compared to combined PTX treatment with radiation (*P*=0.0277). On the other hand, a histopathological study of lung sections revealed no significant difference in histopathological changes between mice treated with PTX and radiation and those treated with NK105 and radiation. Flow-cytometric analysis showed that NK105-treated LLC tumour cells showed more severe arrest at the G2/M phase as compared to PTX-treated tumour cells. The superior radiosensitising activity of NK105 was thus considered to be attributable to the more severe cell cycle arrest at the G2/M phase induced by NK105 as compared to that induced by free PTX. The present study results suggest that further clinical trials are warranted to determine the efficacy and feasibility of combined NK105 therapy with radiation.

Paclitaxel (PTX) has been demonstrated to be one of the most effective anticancer agents available at present (Carney, 1996; Khayat *et al*, 2000). Besides its antitumour activity, its ability to induce radiosensitisation has been reported both *in vitro* (Tishler *et al*, 1992; Choy *et al*, 1993; Lokeshwar *et al*, 1995; Rodriguez *et al*, 1995) and *in vivo* (Milas *et al*, 1994, 1995; Cividalli *et al*, 1998) this effect has been attributed to its effect of stabilising microtubules and inducing cell cycle arrest at the G2/M phase, the most radiosensitive phase of the cell cycle (Terasima and Tolmach, 1963; Sinclair and Morton, 1966). As several clinical studies have demonstrated the efficacy of PTX-based chemotherapy combined with radiotherapy, the combined modality is considered to be a potentially important treatment option for lung and breast cancer (Choy *et al*, 1998a, 1998b, 2000; Dowell *et al*, 1999; Formenti *et al*, 2003; Kao *et al*, 2005).

The adverse effects of radiation, namely, lung toxicities in patients with breast or lung cancer treated by thoracic radiation, are of great concern, and may be dose limiting or even have a negative impact on the quality of life of the patients, even though radiation is an efficient treatment option. Lung toxicities often result in lung fibrosis, necessitating change of the treatment method and causing much distress or even death of the patients (Penney and Rubin, 1977; Early Breast Cancer Trialists' Collaborative Group, 2000; Lind *et al*, 2002). Some clinical trials actually reported an increased incidence of pneumonitis following combined PTX therapy with radiation in patients with breast or lung cancer (Taghian *et al*, 2001; Hanna *et al*, 2002; Chen and Okunieff, 2004).

Although widely used, PTX itself has several adverse effects, such as peripheral sensory neuropathy (Rowinsky *et al*, 1993; Rowinsky and Donehower, 1995), and its poor solubility in water is also associated with such effects as anaphylaxis and other severe hypersensitivity reactions attributable to Cremophor EL and ethanol, which are essential for solubilising PTX (Weiss *et al*, 1990; Rowinsky and Donehower, 1995). In order to overcome these problems, we prepared a new formulation, NK105, which is a PTX-incorporating polymeric micellar nanoparticle (85 nm in size) (Hamaguchi *et al*, 2005). NK105 is formed by facilitating the self-association of amphiphilic block copolymers constructed using polyethylene glycol (PEG) as the hydrophilic segment and modified polyaspartate as the hydrophobic segment in an aqueous medium. Owing to the PEG constituting the outer shell of the micelles, NK105 is soluble in water. In addition, PEG also confers a stealth property to the formulation, that allows the micellar drug preparation to be less avidly taken up by the reticuloendothelial system (RES) and to be retained in the circulation for a longer period of time (Klibanov *et al*, 1990, 1991; Allen, 1994; Gabizon *et al*, 1996). The prolonged circulation time and the ability of NK105 to extravasate through the leaky tumour vasculature (i.e., the EPR (enhanced permeability and retention) effect) causes accumulation of PTX in tumour tissues (Matsumura and Maeda, 1986; Maeda and Matsumura, 1989). We previously demonstrated that NK105 is associated with reduced neurotoxicity and also exerts more potent antitumour activity on human cancer xenograft, as compared to free PTX. In addition, because of its solubility in water, it is expected that the incidence of anaphylaxis and hypersensitivity reactions attributable to Cremophor EL and ethanol may also be reduced with NK105. A clinical trial of NK105 is now under way.

In this context, it is expected that the use of NK105 in place of PTX in combination with radiation may also yield superior results, because of the more potent antitumour activity of this drug as compared to that of free PTX. In this study, we evaluated the antitumour activity and severity of lung fibrosis induced by PTX and NK105 administered in combination with thoracic radiation, to examine whether combined NK105 chemotherapy with radiation would be an acceptable or useful treatment modality.

## MATERIALS AND METHODS

### Mice

Eight-week-old female C57BL/6J mice were purchased from Charles River Japan Inc. (Kanagawa, Japan). All the animal procedures were performed in compliance with the guidelines for the care and use of experimental animals, drawn up by the Committee for Animal Experimentation of the National Cancer Center; these guidelines meet the ethical standards required by law and also comply with the guidelines for the use of experimental animals in Japan.

### PTX and NK105

Paclitaxel was purchased from Merican Corp. (Tokyo, Japan). NK105 is a PTX-incorporating ‘core-shell-type’ polymeric micellar nanoparticle formulation that was prepared by a previously reported procedure (Hamaguchi *et al*, 2005). Briefly, polymeric micellar particles were formed by facilitating the self-association of amphiphilic block copolymers in an aqueous medium. The polymer of NK105 was constructed using PEG as the hydrophilic segment and modified polyaspartate as the hydrophobic segment. The carboxylic groups of the polyaspartate block were modified by the esterification reaction with 4-phenyl-1-butanol, resulting in conversion of half of the groups to 4-phenyl-1-butanolate. Molecular weight of the polymers was determined to be approximately 2000 (PEG block: 12 000; moditied polyaspartate block: 8000).

Via the self-association process, PTX was incorporated into the inner core of the micelle system by physical entrapment through hydrophobic interactions between the drug and specifically well-designed block copolymers for PTX. NK105 was obtained as a freeze-dried formulation and contained ca.23% (WW^−1^) of PTX. Finally, NK105, PTX-incorporating polymeric micellar nanoparticle formulation with a single and narrow size distribution, was obtained. The weight-average diameter of the nanoparticles was approximately 85 nm ranging from 20 to 430 nm.

### Irradiation

The mice were anesthetised by intraperitoneal (i.p.) injection of nembutal (75 mg kg^−1^) and placed on the stage for irradiation. The whole thorax or subcutaneous (s.c.) tumours of the thigh were irradiated using a Faxitron cabinet X-ray system model CP-160 by 100 kV X-rays from a linear accelerator, at a dose rate of 2 Gy min^−1^. Totally 12 Gy was irradiated to each mouse. The whole body except irradiated parts, lung field or tumour lesion, were shielded with specially designed lead blocks.

### Flow cytometry

At 24 h after the injection of PTX or NK105 into the Lewis lung carcinoma (LLC) tumour-bearing C57BL/6j mice, the tumours were excised, minced in PBS, and fixed in 70% ethanol at 4°C for 48 h. After being fixed, the tumours were digested with 0.04% pepsin (Sigma chemical co., St Louis, MO, USA) in 0.1 N HCl for 60 min at 37°C in a shaking bath for preparing single-nuclei suspensions. The nuclei were then centrifuged, washed twice with PBS, and stained with 40 *μ*g ml^−1^ of propidium iodide (Molecular Probes, OR, USA) in the presence of 100 *μ*g ml^−1^ RNase in 1 ml PBS for 30 min at 37°C. The stained nuclei were analysed with a B-D FACSCalibur (BD Biosciences, San Jose, CA, USA). The cell cycle distribution was analysed using the Modfit program (Verity Software House Inc., Topsham, ME, USA).

### Evaluation of the antitumour activity

For this experiment, 3 × 10^6^ LLC cells were inoculated s.c. into the right thighs of mice. The tumour volume was calculated using the formula, tumour volume (mm^3^)=*a* × *b*^2^/2 (*a*=longest tumour diameter, *b*=shortest tumour diameter). When the tumour volume reached approximately 100 mm^3^ on day 14 after the tumour inoculation, the mice were randomly allocated to test groups of about four or five mice each, and started the treatment on the same day. There were six test groups, as follows: untreated control, PTX treatment alone, NK105 treatment alone, radiation alone, combined PTX treatment with radiation, and combined with NK105 treatment with radiation.

In the groups receiving PTX or NK105, the mice were administered a single intravenous (i.v). injection of PTX or NK105 at the dose of 45 mg kg^−1^; 24 h after the drugs were administered, the tumour sites of the mice in the groups scheduled to receive radiation were irradiated.

The antitumour activity of each treatment regimen was evaluated by measuring the tumour volume. Tumour volume and body weight was measured every 3 days.

### Evaluation of lung toxicity

The severity of lung toxicity was evaluated histologically in the following test groups; untreated control (*n*=6), radiation treatment alone (*n*=6), combined PTX treatment with radiation (*n*=9), and combined NK105 treatment with radiation (*n*=10). Mice were administered a single i.v. injection of PTX or NK105 at the dose of 45 mg kg^−1^; 24 h after the drugs were administered, the thorax of the mice in the groups scheduled to receive radiation was irradiated. All the mice were killed 36 weeks after the drug administration. At the time of the killing, the lungs were removed, and the right lungs were fixed in 10% buffered formalin for 24 h, then embedded in paraffin. The lungs were inflated at 20 cm water pressure by intratracheal infusion of 10% buffered formalin before fixation. Sections (5 *μ*m- thick) were stained with haematoxylin and eosin (H&E) and observed under the light microscope. The severity of the pulmonary fibrosis was assessed based on Ashcroft's scoring system (Ashcroft *et al*, 1988). Briefly, all the fields of each lung section were scanned under a Leica microscope at a magnification of × 100, then each field was visually graded from 0 (normal lung) to 8 (total fibrotic obliteration of the field). The mean grades obtained for all of the fields was then calculated as the visual fibrotic score.

### Immunohistochemistry

The lung sections were deparaffinised and rehydrated, then microwaved in 0.01 M sodium citrate buffer for 15 min at 90°C to retrieve epitopes, and cooled at room temperature. An endogenous peroxidase blocking solution of 3% hydrogen peroxide was applied for 20 min at room temperature. After blocking the nonspecific binding sites with 2% normal goat serum, the sections were incubated with rabbit anti-mouse collagen III immunoglobulin G (IgG) (Chemicon International, Temecula, CA, USA) overnight at 4°C. The sections were then washed with PBS, followed by the addition of biotin-conjugated goat anti-rabbit IgG (Vector Laboratories Inc., Burlingame, CA, USA) and incubation for 30 min at room temperature. The sections were then washed and incubated with horseradish-peroxidase-conjugated avidin–biotin complex (Vector Laboratories Inc., Burlingame, CA, USA) at room temperature for 30 min, in accordance with the manufacturer's instructions (Vector Laboratories Inc.). The immunoreactions were visualised using 3,3′-diaminobenzidine as the substrate and counterstaining with haematoxylin.

### Statistical analysis

Date were expressed the mean±s.d. Differences between the test groups were analysed by Student's *t*-test. We used Stat View (SAS Institute Inc.) statistical software. A value of *P*<0.05 was considered statistically significant.

## RESULTS

### Cell cycle analysis

**Figure 1:**
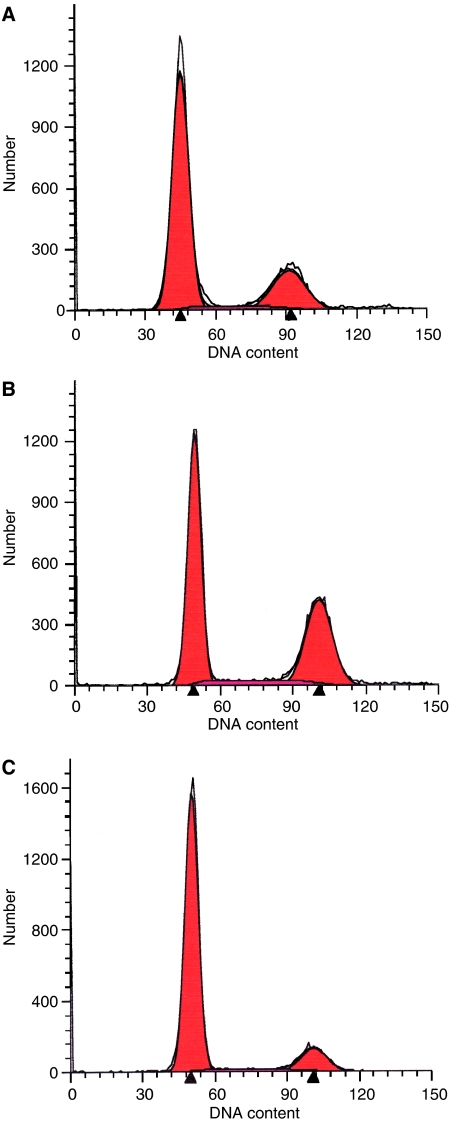
Cell cycle analysis. Cell cycle analysis of LLC tumour cells 24 h after PTX (**A**) or NK105 administration (**B**). Untreated control cells are shown in (**C**).

At 24 h after the administration of PTX or NK105 to the LLC-tumour-bearing mice, severe cell cycle arrest at the G2/M phase was observed in the tumour cells treated with the drugs as compared with that in the control (no drug treatment) (Figure 1C). There was a tendency towards the NK105-treated LLC tumour cells (Figure 1B) showing more severe arrest at the G2/M phase than the PTX-treated cells (Figure 1A).

### Antitumour activity

**Figure 2:**
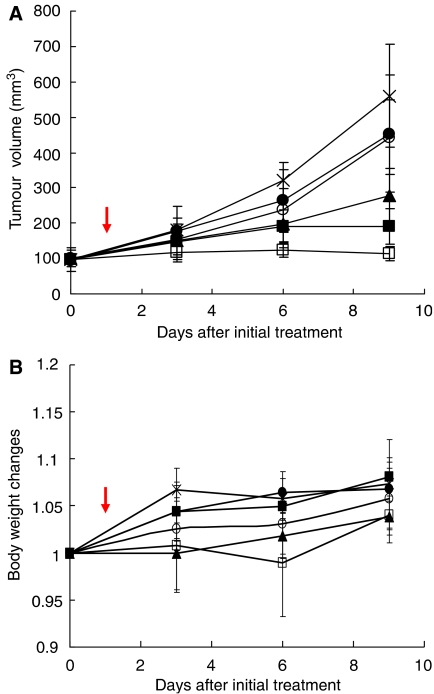
Antitumour activity. Changes in the LLC tumour growth rates in the mice. (**A**) Mice receiving TXL-alone (•), NK105-alone (○), combined treatment with PTX and radiation (▪), and combined treatment with NK105 and radiation (□) were administered a single i.v. injection of PTX or NK105 at the dose 45 mg kg^−1^ on day 14 after the tumour inoculation (=on day 0 after the initial treatment). After 24 h the drugs were administered, the mice in the radiation-alone (▵) and the combined-treatment groups were irradiated (arrow). Mice in the control group (×) were given no treatment. (**B**) Changes in the relative body weight. Data were derived from the same mice as those used in the present study.

Decreased tumour growth rates of the LLC tumours were observed in the mice of the radiation alone, combined PTX with radiation, and combined NK105 with radiation groups. No antitumour activity was observed following treatment with either PTX or NK105 alone, because LLC is primarily a PTX-resistant tumour. Combined NK105 therapy with radiation yielded superior antitumour activity as compared to both radiation alone (*P*=0.0047) and combined PTX therapy with radiation (*P*=0.0277) on the day 9 after the treatment initiation (Figure 2A). No significant differences in body weight changes were noted among the groups tested (Figure 2B).

### Lung toxicities

**Figure 3:**
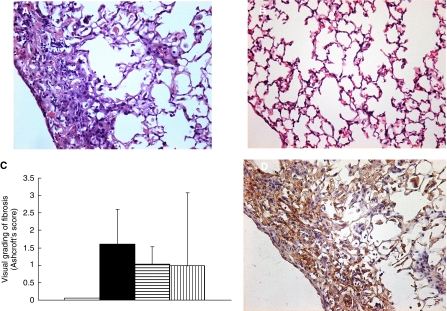
H&E staining of the lungs of C57BL/6J mice surviving 36 weeks after the thoracic radiation (**A**) and sham radiation (**B**). (**C**) Semiquantitative analyses to estimate the severity of pulmonary fibrosis in the mice receiving sham radiation (□), thoracic radiation alone (▪), combined PTX with radiation (), and combined NK105 with radiation (). H&E- stained lung tissue sections were assessed to estimate the severity of pulmonary fibrosis by visual grading of fibrosis (Ashcroft's score). Collagen III staining of the irradiated lungs of mice (**D**).

Histopathological examination of the lung sections of all the mice receiving radiation showed inflammatory cell infiltration, appearance of intra-alveolar macrophages, and destruction of the alveolar architecture. Major portions of the alveolar septa in the lung sections prepared from the irradiated mice showed slight thickening, although no massive structural destruction was observed (Figure 3A). On the other hand, the lung sections prepared from the control nonirradiated group showed no significant histopathological changes (Figure 3B). Ashcroft's fibrosis scores in the groups of mice that received radiation ranged from 0.975 to 1.426, with no significant differences among the groups. The score in the control group was nearly zero. In the groups receiving radiation, the severity of lung fibrosis differed significantly among the mice within the same groups, as did the Ashcroft's scores, that is, the s.d. of the Ashcroft's scores in the mice receiving radiation was very high (Figure 3C).

### Type III collagen deposition

Immunohistochemical analysis of lung sections prepared from the mice receiving radiation revealed significant collagen deposition, especially in the subpleural regions, while that of lung sections prepared from the control group showed little collagen deposition. There were no significant differences among the different groups receiving radiation (Figure 3D).

## DISCUSSION

It is well known that PTX enhances the radiosensitivity of tumour cells by inducing cell cycle arrest at the G2/M phase, the most radiosensitive phase of the cell cycle (Terasima and Tolmach, 1963; Sinclair and Morton, 1966). Many reports have confirmed the radiosensitising effect of PTX in different cell lines (Tishler *et al*, 1992; Choy *et al*, 1993; Lokeshwar *et al*, 1995; Rodriguez *et al*, 1995), *in vivo* experiments (Milas *et al*, 1994, 1995; Cividalli *et al*, 1998), and in several clinical trials of combined PTX with radiation therapy according to different schedules (Dillman *et al*, 1990; Arriagada *et al*, 1991; Morton *et al*, 1991; Furuse *et al*, 1999; Sause *et al*, 2000; Chen *et al*, 2003). Chen *et al* (2003) examined the optimal timing of PTX treatment and irradiation in relation to the cell cycle, and recommended that radiation be given at least 5 h after PTX administration, because G2/M arrest of a lung cancer cell line was shown to start at 4 h after PTX treatment and to last for 44 h.

In our experimental model to evaluate the antitumour activity, the tumours were irradiated 24 h after a single i.v. injection of PTX or NK105. No significant increase in the antitumour activity as compared with that in the control (no treatment) was observed following a single i.v. injection of either PTX or NK105 at the dose of 45 mg kg^−1^; LLC tumours are known to be primarily resistant to PTX. In fact, the IC_50_ of PTX against an LLC tumour cell line was shown to be 84.1 nM, which is about 10-fold higher than that of NK105 against various cancer cell lines tested in our previous work (Hamaguchi *et al*, 2005). Combined NK105 therapy with radiation yielded superior antitumour activity as compared with radiation alone or combined PTX therapy with radiation. This result suggests that NK105 has a more potent radiosensitising effect than PTX. In our study, there was a tendency towards NK105-treated LLC tumour cells showing more severe arrest at the G2/M phase as compared to PTX-treated cells at 24 h after the injection of the drugs, the timing of the radiation treatment, probably because NK105 allows a higher concentration of PTX to be maintained in the tumour than conventional PTX (Hamaguchi *et al*, 2005). We suppose that this is the reason why NK105 exerted more potent radiosensitising activity than PTX.

Next, we were concerned about the adverse effects of combined NK105 therapy with radiation. New micellar drugs are designed based on the idea that DDS can accumulate in the tumour selectively, while showing reduced distribution in normal tissues. We demonstrated that the incorporation of cisplatin into micelles significantly reduced the nephrotoxicity and neurotoxicity of cisplatin (Uchino *et al*, 2005). However, it was also shown that micelle- incorporated cisplatin caused transient liver dysfunction because it was trapped more avidly by the RES as compared to free cisplatin, even though the PEG of the outer shell of the micelle confers the so-called stealth effect.

In this study, our examination of the lung sections of mice treated with NK105 and radiation revealed that the histopathological changes such as inflammatory cell infiltration, appearance of intra-alveolar macrophages, and destruction of the alveolar architecture were induced by thoracic radiation and not by the accumulation of NK105 in the lung. There were no significant differences in the histopathological changes observed among the mice treated by NK105 and radiation and mice treated by radiation alone or PTX with radiation. The severity of lung fibrosis did not differ significantly among the test groups either. Although some clinical trials reported an increased incidence of pneumonitis and esophagitis following combined PTX therapy with radiation (Taghian *et al*, 2001; Hanna *et al*, 2002; Chen and Okunieff, 2004), others reported no influence on the incidence of such adverse effects (Ellerbroek *et al*, 2003; Yu *et al*, 2003). Several clinical trials and *in vivo* experiments have discussed the subject, however, no definitive conclusion has been arrived at (Mason *et al*, 1995; Choy *et al*, 1998; Yu *et al*, 2004; Kao *et al*, 2005). In our study, in regard to the incidence of esophagitis, there were no significant differences in the histopathological changes observed in the esophageal sections at one week after the treatment among the test groups (data not shown).

In conclusion, we demonstrated that combined NK105 chemotherapy with radiation exerts significant antitumour activity. Furthermore, the lung toxicity of this combined treatment modality was also acceptable as compared with that observed following radiation alone or combined PTX therapy with radiation. However, further studies are necessary to determine the effectiveness of NK105 in terms of its radiosensitising effect.

## References

[bib1] Allen TM (1994) Long-circulating (sterically stabilized) liposomes for targeted drug delivery. Trends Pharmacol Sci 15: 215–220 7940982 10.1016/0165-6147(94)90314-x

[bib2] Arriagada R, Le Chevalier T, Quoix E, Ruffie P, de Cremoux H, Douillard JY, Tarayre M, Pignon JP, Laplanche A (1991) ASTRO (American Society for Therapeutic Radiology and Oncology) plenary: Effect of chemotherapy on locally advanced non-small cell lung carcinoma: a randomized study of 353 patients. GETCB (Groupe d'Etude et Traitement des Cancers Bronchiques), FNCLCC (Federation Nationale des Centres de Lutte contre le Cancer) and the CEBI trialists. Int J Radiat Oncol Biol Phys 20: 1183–1190 1646194 10.1016/0360-3016(91)90226-t

[bib3] Ashcroft T, Simpson JM, Timbrell V (1988) Simple method of estimating severity of pulmonary fibrosis on a numerical scale. J Clin Pathol 41: 467–470 3366935 10.1136/jcp.41.4.467 PMC1141479

[bib4] Carney DN (1996) Chemotherapy in the management of patients with inoperable non-small cell lung cancer. Semin Oncol 23: 71–75 9007127

[bib5] Chen Y, Okunieff P (2004) Radiation and third-generation chemotherapy. Hematol Oncol Clin North Am 18: 55–80 15005281 10.1016/s0889-8588(03)00145-x

[bib6] Chen Y, Pandya K, Keng PC, Johnstone D, Li J, Lee YJ, Smudzin T, Okunieff P (2003) Phase I/II clinical study of pulsed paclitaxel radiosensitization for thoracic malignancy: a therapeutic approach on the basis of preclinical research of human cancer cell lines. Clin Cancer Res 9: 969–975 12631594

[bib7] Choy H, Akerley W, Safran H, Graziano S, Chung C, Williams T, Cole B, Kennedy T (1998a) Multiinstitutional phase II trial of paclitaxel, carboplatin, and concurrent radiation therapy for locally advanced non-small-cell lung cancer. J Clin Oncol 16: 3316–3322 9779707 10.1200/JCO.1998.16.10.3316

[bib8] Choy H, Devore III RF, Hande KR, Porter LL, Rosenblatt P, Yunus F, Schlabach L, Smith C, Shyr Y, Johnson DH (2000) A phase II study of paclitaxel, carboplatin, and hyperfractionated radiation therapy for locally advanced inoperable non-small-cell lung cancer (a Vanderbilt Cancer Center Affiliate Network Study). Int J Radiat Oncol Biol Phys 47: 931–937 10863062 10.1016/s0360-3016(00)00420-x

[bib9] Choy H, Rodriguez FF, Koester S, Hilsenbeck S, Von Hoff DD (1993) Investigation of taxol as a potential radiation sensitizer. Cancer 71: 3774–3778 8098270 10.1002/1097-0142(19930601)71:11<3774::aid-cncr2820711147>3.0.co;2-0

[bib10] Choy H, Safran H, Akerley W, Graziano SL, Bogart JA, Cole BF (1998b) Phase II trial of weekly paclitaxel and concurrent radiation therapy for locally advanced non-small cell lung cancer. Clin Cancer Res 4: 1931–1936 9717821

[bib11] Cividalli A, Arcangeli G, Cruciani G, Livdi E, Cordelli E, Danesi DT (1998) Enhancement of radiation response by paclitaxel in mice according to different treatment schedules. Int J Radiat Oncol Biol Phys 40: 1163–1170 9539573 10.1016/s0360-3016(97)00912-7

[bib12] Dillman RO, Seagren SL, Propert KJ, Guerra J, Eaton WL, Perry MC, Carey RW, Frei III EF, Green MR (1990) A randomized trial of induction chemotherapy plus high-dose radiation *vs* radiation alone in stage III non-small-cell lung cancer. N Engl J Med 323: 940–945 2169587 10.1056/NEJM199010043231403

[bib13] Dowell JE, Sinard R, Yardley DA, Aviles V, Machtay M, Weber RS, Weinstein GS, Chalian AA, Carbone DP, Rosenthal DI (1999) Seven-week continuous-infusion paclitaxel concurrent with radiation therapy for locally advanced non-small cell lung and head and neck cancers. Semin Radiat Oncol 9: 97–101 10210547

[bib14] Early Breast Cancer Trialists' Collaborative Group (2000) Favourable and unfavourable effects on long-term survival of radiotherapy for early breast cancer: an overview of the randomised trials. Early Breast Cancer Trialists' Collaborative Group. Lancet 355: 1757–1770 10832826

[bib15] Ellerbroek N, Martino S, Mautner B, Tao ML, Rose C, Botnick L (2003) Breast-conserving therapy with adjuvant paclitaxel and radiation therapy: feasibility of concurrent treatment. Breast J 9: 74–78 12603378 10.1046/j.1524-4741.2003.09203.x

[bib16] Formenti SC, Volm M, Skinner KA, Spicer D, Cohen D, Perez E, Bettini AC, Groshen S, Gee C, Florentine B, Press M, Danenberg P, Muggia F (2003) Preoperative twice-weekly paclitaxel with concurrent radiation therapy followed by surgery and postoperative doxorubicin-based chemotherapy in locally advanced breast cancer: a phase I/II trial. J Clin Oncol 21: 864–870 12610186 10.1200/JCO.2003.06.132

[bib17] Furuse K, Fukuoka M, Kawahara M, Nishikawa H, Takada Y, Kudoh S, Katagami N, Ariyoshi Y (1999) Phase III study of concurrent *vs* sequential thoracic radiotherapy in combination with mitomycin, vindesine, and cisplatin in unresectable stage III non-small-cell lung cancer. J Clin Oncol 17: 2692–2699 10561343 10.1200/JCO.1999.17.9.2692

[bib18] Gabizon A, Chemla M, Tzemach D, Horowitz AT, Goren D (1996) Liposome longevity and stability in circulation: effects on the *in vivo* delivery to tumors and therapeutic efficacy of encapsulated anthracyclines. J Drug Target 3: 391–398 8866658 10.3109/10611869608996830

[bib19] Hamaguchi T, Matsumura Y, Suzuki M, Shimizu K, Goda R, Nakamura I, Nakatomi I, Yokoyama M, Kataoka K, Kakizoe T (2005) NK105, a paclitaxel-incorporating micellar nanoparticle formulation, can extend *in vivo* antitumour activity and reduce the neurotoxicity of paclitaxel. Br J Cancer 92: 1240–1246 15785749 10.1038/sj.bjc.6602479 PMC2361981

[bib20] Hanna YM, Baglan KL, Stromberg JS, Vicini FA, A Decker D (2002) Acute and subacute toxicity associated with concurrent adjuvant radiation therapy and paclitaxel in primary breast cancer therapy. Breast J 8: 149–153 12047471 10.1046/j.1524-4741.2002.08306.x

[bib21] Kao J, Conzen SD, Jaskowiak NT, Song DH, Recant W, Singh R, Masters GA, Fleming GF, Heimann R (2005) Concomitant radiation therapy and paclitaxel for unresectable locally advanced breast cancer: results from two consecutive phase I/II trials. Int J Radiat Oncol Biol Phys 61: 1045–1053 15752883 10.1016/j.ijrobp.2004.07.714

[bib22] Khayat D, Antoine EC, Coeffic D (2000) Taxol in the management of cancers of the breast and the ovary. Cancer Invest 18: 242–260 10754992 10.3109/07357900009031828

[bib23] Klibanov AL, Maruyama K, Beckerleg AM, Torchilin VP, Huang L (1991) Activity of amphipathic poly(ethylene glycol) 5000 to prolong the circulation time of liposomes depends on the liposome size and is unfavorable for immunoliposome binding to target. Biochim Biophys Acta 1062: 142–148 2004104 10.1016/0005-2736(91)90385-l

[bib24] Klibanov AL, Maruyama K, Torchilin VP, Huang L (1990) Amphipathic polyethyleneglycols effectively prolong the circulation time of liposomes. FEBS Lett 268: 235–237 2384160 10.1016/0014-5793(90)81016-h

[bib25] Lind PA, Marks LB, Hardenbergh PH, Clough R, Fan M, Hollis D, Hernando ML, Lucas D, Piepgrass A, Prosnitz LR (2002) Technical factors associated with radiation pneumonitis after local +/− regional radiation therapy for breast cancer. Int J Radiat Oncol Biol Phys 52: 137–143 11777631 10.1016/s0360-3016(01)01715-1

[bib26] Lokeshwar BL, Ferrell SM, Block NL (1995) Enhancement of radiation response of prostatic carcinoma by taxol: therapeutic potential for late-stage malignancy. Anticancer Res 15: 93–98 7733649

[bib27] Maeda H, Matsumura Y (1989) Tumoritropic and lymphotropic principles of macromolecular drugs. Crit Rev Ther Drug Carrier Syst 6: 193–210 2692843

[bib28] Mason KA, Milas L, Peters LJ (1995) Effect of paclitaxel (taxol) alone and in combination with radiation on the gastrointestinal mucosa. Int J Radiat Oncol Biol Phys 32: 1381–1389 7635778 10.1016/0360-3016(95)00037-Y

[bib29] Matsumura Y, Maeda H (1986) A new concept for macromolecular therapeutics in cancer chemotherapy: mechanism of tumoritropic accumulation of proteins and the antitumor agent smancs. Cancer Res 46: 6387–6392 2946403

[bib30] Milas L, Hunter NR, Mason KA, Kurdoglu B, Peters LJ (1994) Enhancement of tumour radioresponse of a murine mammary carcinoma by paclitaxel. Cancer Res 54: 3506–3510 7912167

[bib31] Milas L, Hunter NR, Mason KA, Milross CG, Saito Y, Peters LJ (1995) Role of reoxygenation in induction of enhancement of tumour radioresponse by paclitaxel. Cancer Res 55: 3564–3568 7627965

[bib32] Morton RF, Jett JR, McGinnis WL, Earle JD, Therneau TM, Krook JE, Elliott TE, Mailliard JA, Nelimark RA, Maksymiuk AW (1991) Thoracic radiation therapy alone compared with combined chemoradiotherapy for locally unresectable non-small cell lung cancer. A randomized, phase III trial. Ann Intern Med 115: 681–686 1656827 10.7326/0003-4819-115-9-681

[bib33] Penney DP, Rubin P (1977) Specific early fine structural changes in the lung irradiation. Int J Radiat Oncol Biol Phys 2: 1123–1132 599063 10.1016/0360-3016(77)90119-5

[bib34] Rodriguez M, Sevin BU, Perras J, Nguyen HN, Pham C, Steren AJ, Koechli OR, Averette HE (1995) Paclitaxel: a radiation sensitizer of human cervical cancer cells. Gynecol Oncol 57: 165–169 7729728 10.1006/gyno.1995.1119

[bib35] Rowinsky EK, Chaudhry V, Forastiere AA, Sartorius SE, Ettinger DS, Grochow LB, Lubejko BG, Cornblath DR, Donehower RC (1993) Phase I and pharmacologic study of paclitaxel and cisplatin with granulocyte colony-stimulating factor: neuromuscular toxicity is dose-limiting. J Clin Oncol 11: 2010–2020 7692001 10.1200/JCO.1993.11.10.2010

[bib36] Rowinsky EK, Donehower RC (1995) Paclitaxel (taxol). N Engl J Med 332: 1004–1014 7885406 10.1056/NEJM199504133321507

[bib37] Sause W, Kolesar P, Taylor SI, Johnson D, Livingston R, Komaki R, Emami B, Curran Jr W, Byhardt R, Dar AR, Turrisi III A (2000) Final results of phase III trial in regionally advanced unresectable non-small cell lung cancer: Radiation Therapy Oncology Group, Eastern Cooperative Oncology Group, and Southwest Oncology Group. Chest 117: 358–364 10669675 10.1378/chest.117.2.358

[bib38] Sinclair WK, Morton RA (1966) X-ray sensitivity during the cell generation cycle of cultured Chinese hamster cells. Radiat Res 29: 450–474 5924188

[bib39] Taghian AG, Assaad SI, Niemierko A, Kuter I, Younger J, Schoenthaler R, Roche M, Powell SN (2001) Risk of pneumonitis in breast cancer patients treated with radiation therapy and combination chemotherapy with paclitaxel. J Natl Cancer Inst 93: 1806–1811 11734597 10.1093/jnci/93.23.1806

[bib40] Terasima T, Tolmach LJ (1963) X-ray sensitivity and DNA synthesis in synchronous populations of HeLa cells. Science 140: 490–492 13980636 10.1126/science.140.3566.490

[bib41] Tishler RB, Geard CR, Hall EJ, Schiff PB (1992) Taxol sensitizes human astrocytoma cells to radiation. Cancer Res 52: 3495–3497 1350755

[bib42] Uchino H, Matsumura Y, Negishi T, Koizumi F, Hayashi T, Honda T, Nishiyama N, Kataoka K, Naito S, Kakizoe T (2005) Cisplatin-incorporating polymeric micelles (NC-6004) can reduce nephrotoxicity and neurotoxicity of cisplatin in rats. Br J Cancer 93: 678–687 16222314 10.1038/sj.bjc.6602772 PMC2361620

[bib43] Weiss RB, Donehower RC, Wiernik PH, Ohnuma T, Gralla RJ, Trump DL, Baker Jr JR, Van Echo DA, Von Hoff DD, Leyland-Jones B (1990) Hypersensitivity reactions from taxol. J Clin Oncol 8: 1263–1268 1972736 10.1200/JCO.1990.8.7.1263

[bib44] Yu TK, Whitman GJ, Thames HD, Strom E, McNeese MD, Perkins GH, Schechter N, Kau S, Buzdar AU, Hortobagyi GN, Thomas E, Buchholz TA (2003) Clinically-relevant pneumonitis is not increased in breast cancer patients treated with sequential paclitaxel and radiation. Int J Radiat Oncol Biol Phys 57(2 Suppl): S127–S128

[bib45] Yu TK, Whitman GJ, Thames HD, Buzdar AU, Strom EA, Perkins GH, Schechter NR, McNeese MD, Kau SW, Thomas ES, Hortobagyi GN, Buchholz TA (2004) Clinically relevant pneumonitis after sequential paclitaxel-based chemotherapy and radiotherapy in breast cancer patients. J Natl Cancer Inst 96: 1676–1681 15547180 10.1093/jnci/djh315

